# High TRIM28 Expression Defines an Aggressive, Immune-Cold Phenotype with Worse Survival Outcomes in ERα-Positive Breast Cancer

**DOI:** 10.3390/biomedicines14071523

**Published:** 2026-07-07

**Authors:** Rashed Alhammad, Najla Salama, Lujain Alhammad

**Affiliations:** 1 Department of Pharmacology, Faculty of Medicine, Kuwait University, Kuwait City 13110, Kuwait; 2 Department of Pharmaceutical Technology, College of Medical Science and Technologies, Tripoli P.O. Box 13628, Libya; 3 Neurology Department, Ibn Sina Hospital, Ministry of Health, Kuwait City 70035, Kuwait; 4 Neuroscience Department, King Faisal Specialist Hospital and Research Centre, Riyadh 12713, Saudi Arabia

**Keywords:** TRIM28, bioinformatics, breast cancer, ERα, survival, metastasis, prognostic biomarker

## Abstract

**Background**: Although ERα-positive breast cancer represents approximately 70% of all breast cancer diagnoses worldwide, specific prognostic biomarkers for this subtype that are capable of stratifying patients remain limited. TRIM28 (KAP1/TIF1β), which is a multifunctional E3 ubiquitin ligase and transcriptional coregulator of ERα, has been shown to play oncogenic roles in multiple malignancies. However, its prognostic significance in ERα-positive breast cancer subtype has not been explored across independent patient cohorts. **Methods**: Multiple bioinformatics tools were employed to assess TRIM28 mRNA expression and prognostic significance across independent patient cohorts totaling over 4000 patients. The Kaplan–Meier plotter was used to examine associations between TRIM28 expression and survival outcomes in ERα-positive breast cancer. Multivariate Cox proportional hazards regression was performed in the METABRIC dataset (n = 1356) to confirm independent prognostic value, with sensitivity analyses using alternative TRIM28 expression cutoffs and PAM50 subtype-specific subgroup analyses. Independent validation was performed using multivariate Cox regression in the TCGA-BRCA Firehose Legacy ERα-positive cohort (n = 372). Gene Set Cancer Analysis (GSCA) was used to investigate pathways associated with TRIM28-correlated genes, and Breast Cancer Gene-Expression Miner (Bc-GenExminer) was used to assess immune cell infiltration. **Results**: TRIM28 expression is significantly elevated in ERα-positive breast cancer compared to normal breast tissue. High TRIM28 expression (defined as the upper quartile, ≥75th percentile, of TRIM28 expression) is independently associated with worse overall survival after adjustment for tumour size, tumour mutational burden (TMB), hormone therapy status, and age, with stratification on histologic grade (HR = 1.21, 95% CI: 1.03–1.41, *p* = 0.0194; METABRIC, n = 1356). This finding was independently validated using multivariate Cox proportional hazards regression in the TCGA-BRCA Firehose Legacy ERα-positive cohort (n = 372; events = 56), in which high TRIM28 expression remained independently associated with worse OS after adjustment for age, T-stage, and TMB (HR = 2.02, 95% CI: 1.10–3.71, *p* = 0.024). PAM50 subtype-specific analyses within METABRIC additionally confirmed an independent association of high TRIM28 with worse OS in Luminal A (HR = 1.37, 95% CI: 1.08–1.74, *p* = 0.009), with a directionally consistent but non-significant trend in Luminal B (HR = 1.21, 95% CI: 0.93–1.58, *p* = 0.155). The prognostic effect was robust across alternative TRIM28 expression cutoffs. TRIM28 expression positively correlates with tumour size, histologic grade, Nottingham Prognostic Index, and TMB, and negatively correlates with immune cell infiltration and is significantly lower in patients receiving hormone therapy. Genes co-expressed with TRIM28 are enriched in cell cycle and DNA damage response activation signatures and RAS/MAPK and RTK pathway inhibition signatures. **Conclusions**: TRIM28 is an independent prognostic biomarker in ERα-positive breast cancer, validated across multiple independent cohorts. These findings nominate TRIM28 as a priority candidate for prospective clinical validation and targeted experimental investigation in ERα-positive breast cancer.

## Simple Summary

TRIM28 is a protein that plays important roles in cancer development, but its significance in oestrogen receptor-positive (ERα-positive) breast cancer—the most common breast cancer subtype—has not been thoroughly investigated. In this study, we analysed data from over 4000 breast cancer patients across multiple independent datasets and found that patients with high TRIM28 levels have significantly worse survival outcomes compared to those with low TRIM28 levels. We also found that high TRIM28 is associated with more aggressive tumour features, a suppressed immune environment within the tumour, and resistance to the chemotherapy drug docetaxel. These findings suggest that TRIM28 could serve as a useful marker to identify ERα-positive breast cancer patients who are at higher risk of poor outcomes, and that targeting TRIM28 may represent a promising strategy for future therapeutic development in this patient group.

## 1. Introduction

Breast cancer is the most commonly diagnosed malignancy and the leading cause of cancer-related death among women worldwide. According to GLOBOCAN 2022 estimates, approximately 2.3 million new cases of female breast cancer and around 666,000 deaths were recorded globally in 2022, accounting for roughly 23.8% of all new cancer cases and 15.4% of all cancer deaths in women [1]. Breast cancer is the most frequently diagnosed cancer in women in 157 of 185 countries and the leading cause of cancer death in women in 111 countries, and the global burden is projected to exceed 6 million new cases and 3 million deaths annually by 2050 [1]. Breast cancer is a biologically heterogeneous disease, and approximately 70% of cases are oestrogen receptor alpha (ERα)-positive, a subtype whose growth is driven by oestrogen signalling and which is typically managed with endocrine (hormone) therapy [2]. Despite the generally favourable prognosis of ERα-positive disease relative to other subtypes, a substantial proportion of patients experience recurrence, metastasis, or resistance to endocrine therapy; consequently, there remains a pressing need for robust prognostic biomarkers capable of stratifying ERα-positive patients according to their risk of adverse outcomes [3].

Tripartite motif-containing 28 (TRIM28), also known as Krüppel-associated box (KRAB)-associated protein 1 (KAP1) or transcriptional intermediary factor 1β (TIF1β), is a member of the TRIM protein family [4,5]. The TRIM family encompasses over 80 members [6], which are implicated in diverse cellular processes and signalling pathways [7]. The TRIM28 gene, which is located on chromosome 19 and spans 6652 base pairs, encodes the TRIM28 protein [8]. TRIM28 protein plays a critical role in numerous biological functions, including transcriptional regulation, DNA damage response, maintenance of stem cell pluripotency, suppression of p53 activity via its intrinsic E3 ubiquitin ligase activity, modulation of apoptosis, and regulation of epithelial-to-mesenchymal transition (EMT) [9,10,11].

Given that TRIM28 overexpression has been demonstrated to enhance cancer proliferation and metastasis across various malignancies, including lung, prostate, and gastric cancers [10,12,13], and that TRIM28 acts as a direct coregulator of ERα in breast cancer [14], evaluating the prognostic significance of TRIM28 expression in ERα-positive breast cancer warrants systematic investigation. Furthermore, given the heterogeneity of breast cancer and the pressing need for novel biomarkers to predict clinical outcomes and optimise personalised therapeutic strategies, coupled with the limited data on TRIM28’s role as a prognostic biomarker in ERα-positive breast cancer, this study aimed to investigate the relevance of TRIM28 in ERα-positive breast cancer through the application of multiple bioinformatics tools.

Although TRIM28 has been shown to promote breast cancer metastasis by stabilising TWIST1 and by reducing the stability of BRD7 [15,16], the role of TRIM28 in the ERα-positive breast cancer subtype remains to be explored. In response to the limited understanding of TRIM28’s potential as a biomarker in ERα-positive breast cancer, an extensive bioinformatics analysis was conducted using multiple databases to evaluate its prognostic relevance. Specifically, we investigated the significance of TRIM28 expression in ERα-positive breast cancer through bioinformatics analysis of clinical indicators and survival data from Bc-GenExMiner and TIMER2.0. The Kaplan–Meier plotter was employed to examine the association between TRIM28 expression and various prognostic indicators, while the GSCA web server was utilised to elucidate the pathways associated with genes correlated with TRIM28. Additionally, the cBio Cancer Genomics Portal was explored to analyse the relationship between TRIM28 expression and clinical parameters, such as tumour size, neoplasm histologic grade, and Nottingham Prognostic Index. Furthermore, the Bc-GenExMiner web server was used to investigate immune cell infiltration in relation to TRIM28 expression in ERα-positive breast cancer. Lastly, multivariate Cox proportional hazards regression was performed across the METABRIC and TCGA-BRCA datasets, comprising over 4000 patients, to confirm independent prognostic value.

## 2. Materials and Methods

### 2.1. Exploring TRIM28 mRNA Expression in Breast Cancer, Normal Tissue, and Tumour-Adjacent Tissue

Two different databases were used to identify the expression of TRIM28 in ERα-positive breast cancer, ERα-negative breast cancer, tumour-adjacent tissue, and normal breast tissue. A mining tool of published annotated genomic data (Bc-GenExminer version 4.1) was used to assess the expression of TRIM28 in ERα-positive breast cancer (n = 530), ERα-negative breast cancer (n = 187), tumour-adjacent tissue (n = 89), and normal breast tissue (n = 92) (http://bcgenex.ico.unicancer.fr/BC-GEM/GEM-Accueil.php?js=1) (accessed on the 12 October 2025) [17]. Expression values in Bc-GenExMiner are reported as log2-transformed microarray signal intensities, standardised within the platform. TRIM28 mRNA expression was also assessed in breast cancer tissue (n = 1093) and in normal breast tissue (n = 112) using the TIMER2.0 database (http://timer.cistrome.org/) (accessed on the 12 October 2025) [18]. TIMER2.0 reports TIMER-normalised expression values derived from RNA-seq data, corrected for tumour purity.

### 2.2. Kaplan–Meier Survival Curve Analysis

The prognostic value of TRIM28 in ERα-positive breast cancer was assessed using the Kaplan–Meier plotter website (http://kmplot.com/analysis/) (accessed on the 12 October 2025) [19]. The following parameters were selected to generate Kaplan–Meier plots in ERα-positive breast cancer: post-progression survival (PPS), overall survival (OS), distant metastases-free survival (DMFS), and relapse-free survival (RFS); probe set option: all probe sets per gene; split patients by: upper quartile; follow-up threshold: 240 months [20,21]. Log-rank *p*-values of <0.05 were considered statistically significant [22,23].

### 2.3. The Correlation Between TRIM28 mRNA Expression with Tumour Size, Neoplasm Histologic Grade, Nottingham Prognostic Index, Hormone Therapy, and Tumour Mutational Burden (TMB) in ERα-Positive Breast Cancer Patients

The METABRIC dataset was downloaded from cBio Cancer Genomics Portal (MA, USA) (http://cbioportal.org) (accessed on the 12 October 2025) Table S1 [24], which contains 2051 records in total. The following exclusion criteria were applied: samples with missing ERα status (n = 32) and ERα-negative patients (n = 484) were excluded, yielding 1535 ERα-positive breast cancer patients for analysis. For the multivariate Cox regression analysis, patients with missing data in any model covariate were additionally excluded (n = 179), yielding a final analytical dataset of 1356 patients (791 events) as illustrated in the patient flow diagram (Supplementary Figure S1). In the ERα-positive cohort, the distribution of TRIM28 mRNA, neoplasm histologic grades, patients receiving hormone therapy, tumour size, Nottingham Prognostic Index, and TMB were explored. In the METABRIC study, TRIM28 mRNA expression levels from cBioPortal are shown as z-scores, which are calculated relative to diploid tumour samples. TRIM28 mRNA expression levels were considered high if their z-scores ≥ 75th percentile of the distribution [25].

### 2.4. The Correlation Between TRIM28 mRNA and the Infiltration of Immune Cells in ERα-Positive Breast Cancer

The Bc-GenExMiner website was used to assess the Pearson’s correlation coefficients between TRIM28 mRNA and several gene markers of immune cells in ERα-positive breast cancer [17]. Various gene markers of immune cells were investigated in the analysis, such as gene markers for T cells, B cells, monocytes, neutrophils, and dendritic cells. A *p*-value of <0.0001 was considered significant.

### 2.5. Identifying the Top Genes That Correlate Significantly with TRIM28 in ERα-Positive Breast Cancer

Bc-GenExMiner was utilised to identify genes that positively and significantly correlate with TRIM28 mRNA in ERα-positive breast cancer, in which genes with >0.5 Pearson’s correlation coefficients and *p*-value of <0.0001 were selected.

### 2.6. The Association of Genes That Correlate with TRIM28 in ERα-Positive Breast Cancer with Signalling Pathways

The enrichment of genes that correlate with TRIM28 in ERα-positive breast cancer in cancer-related pathway inhibition and activation signatures, including apoptosis, cell cycle, and PI3K/AKT, were investigated using the GSCA website (Houston, TX, USA) (https://guolab.wchscu.cn/GSCA/#/) (accessed on the 12 October 2025) [26]. In the analysis, breast-invasive carcinoma patients with available data and paired samples (paired tumour–normal tissue) were included.

### 2.7. Identifying the Correlation Between TRIM28 and the IC_50_ of Different Cancer Drugs

The correlation between the expression of TRIM28 and the IC_50_ of different cancer drugs in the Genome of Drug Sensitivity in Cancer (GDSC) database was explored using the GSCA website (Houston, TX, USA) (https://guolab.wchscu.cn/GSCA/#/) (accessed on the 12 October 2025) [26].

### 2.8. Statistical Analysis

GraphPad Prism version 9.0.0 (GraphPad Software, San Diego, CA, USA; www.graphpad.com) was used to generate graphical representations of the data. The significance of the difference between more than two groups was assessed using the Kruskal–Wallis test, whereas the significance of the difference between two groups was assessed using the Mann–Whitney test. Additionally, Benjamini–Hochberg false discovery rate (FDR) correction was used and FDR-adjusted q-values were reported alongside raw *p*-values for multiple comparisons. The Shapiro–Wilk test prior to statistical test selection was used to assess the normality of data distribution. Given non-normal distribution across all groups (Shapiro–Wilk *p* < 0.05 in all cases), non-parametric tests were used throughout. The Kruskal–Wallis test was used for three or more independent groups, while the Mann–Whitney U test was used for comparisons between two independent groups.

### 2.9. Multivariate Cox Proportional Hazards Regression Analysis

To assess whether TRIM28 expression constitutes an independent prognostic factor for OS in ERα-positive breast cancer, multivariate Cox proportional hazards regression analysis was performed using the METABRIC dataset. The survival package in R (version 3.5.1) was used to conduct all analyses. The same upper quartile was selected as the cutoff to be consistent with the approach applied in the KMplot survival analysis, in which patients were considered high TRIM28 if their z-scores ≥ 75th percentile of the distribution (cutoff z-score = 0.614). Patients with missing data in any model variable were excluded from the analysis. Schoenfeld residuals (cox.zph) were used to assess the proportional hazards (PH) assumption for each covariate. The primary multivariate model adjusted for tumour size, TMB, hormone therapy status, and age at diagnosis (continuous, years), with stratification on histologic grade to address PH violation. Because Schoenfeld residuals indicated a PH violation for age when modelled as a continuous covariate, a sensitivity analysis was additionally performed in which age was stratified as tertiles alongside grade; this resolved the PH violation and yielded a consistent result for TRIM28 (Supplementary Table S2). Nottingham Prognostic Index (NPI) was excluded from the primary multivariate model because it is mathematically derived from tumour size, lymph node stage, and histological grade (NPI = 0.2 × tumour size + lymph node stage + grade), and its inclusion alongside its constituent variables produces multicollinearity that distorts the coefficient estimate for grade. Sensitivity analyses were performed using (i) alternative TRIM28 dichotomisation thresholds (median, tertile, upper quartile, top decile); (ii) TRIM28 modelled as a continuous z-score variable; (iii) PAM50 subtype-specific subgroup analyses (Luminal A and Luminal B), each adjusted for tumour size, TMB, and hormone therapy status; and (iv) the original Cox model specification including NPI alongside its component variables, retained for completeness and to demonstrate the robustness of the TRIM28 effect across model specifications. Hazard ratios, 95% confidence intervals, and *p*-values were reported for all models, and the concordance statistic (C-index) was used to assess model discriminatory performance.

### 2.10. Independent Validation Using Multivariate Cox Regression in TCGA-BRCA Firehose Legacy

For independent validation of the prognostic association of TRIM28 with overall survival, the TCGA-BRCA Firehose Legacy dataset was downloaded from the cBio Cancer Genomics Portal (http://cbioportal.org). TRIM28 mRNA expression was obtained as RNA-seq z-scores relative to diploid samples. Inclusion criteria were: ERα-positive disease (by IHC), available TRIM28 mRNA expression data, complete OS follow-up (OS time and event status), and OS time > 0 months. Patients with missing data in any model covariate were excluded from the multivariate analysis. The final analytical cohort comprised 372 patients with 56 events. TRIM28 expression was dichotomised at the upper quartile (z-score ≥ 75th percentile of the cohort distribution; cutoff = 0.807) to maintain methodological consistency with the METABRIC primary analysis. Multivariate Cox proportional hazards regression was performed using the survival package in R, with adjustment for age at diagnosis (continuous), American Joint Committee on Cancer T-stage (categorical: T1, T2, T3, T4), and TMB (continuous). Histologic grade was not available for TCGA-BRCA patients and could therefore not be included as a covariate. Hazard ratios, 95% confidence intervals, and *p*-values were derived from the multivariate Cox model. Sensitivity analyses across alternative TRIM28 dichotomisation cutoffs (median, tertile, upper quartile, top decile) were performed to assess the robustness of the prognostic signal. Univariate Cox regression results are also reported for comparison.

## 3. Results

Table 1 summarises the demographic and clinical characteristics of all the patient cohorts used in this study.

### 3.1. Higher TRIM28 mRNA Was Observed in ERα-Positive Breast Cancer Patients Compared to ERα-Negative Breast Cancer Patients, Tumour-Adjacent Tissue, and Normal Breast Tissue

**Figure 1:**
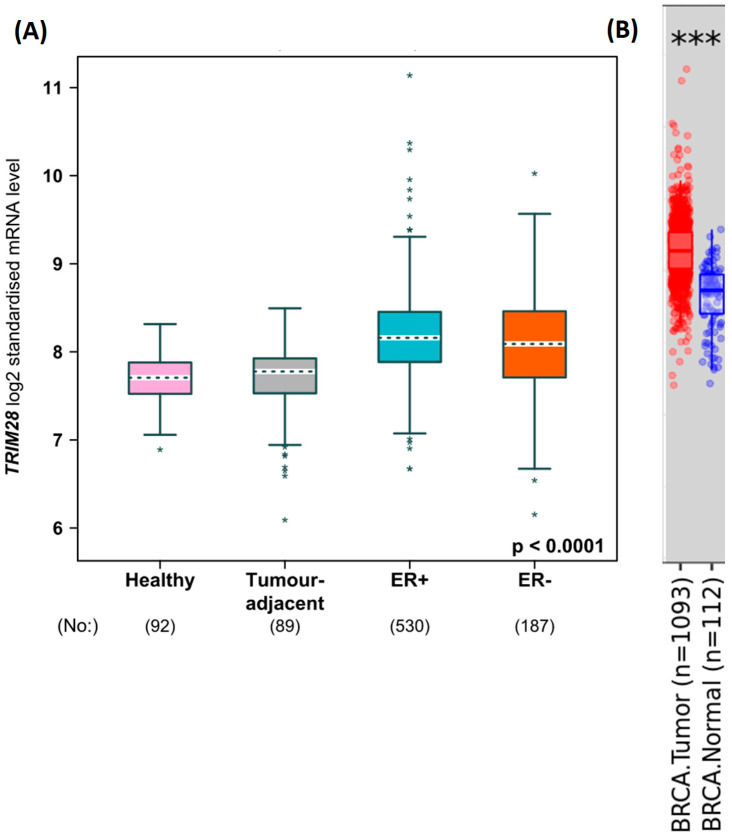
TRIM28 mRNA expression in breast cancer and normal tissues. (**A**) TRIM28 mRNA expression in normal breast tissue (n = 92), tumour-adjacent tissue (n = 89), ERα-negative breast cancer (n = 187), and ERα-positive breast cancer (n = 530) using Bc-GenExMiner v4.1. (**B**) TRIM28 mRNA expression in breast cancer tissue (n = 1093) and normal breast tissue (n = 112) using TIMER2.0. * *p* < 0.05; *** *p* < 0.001.

Several bioinformatics platforms, such as TIMER2.0 and Bc-GenExMiner, were utilised to assess the mRNA expression of TRIM28 in breast cancer versus normal breast tissue. Significantly higher TRIM28 mRNA was observed in ERα-positive breast cancer tissue (n = 530) and ERα-negative breast cancer tissue (n = 187) compared to normal breast tissue (n = 92) (Figure 1A). Moreover, significantly higher TRIM28 mRNA expression was observed in breast cancer tissue (n = 1093) compared to normal tissue (n = 112) (Figure 1B).

### 3.2. TRIM28 mRNA Positively Correlates with Poor Prognosis in ERα-Positive Breast Cancer Patients

**Figure 2:**
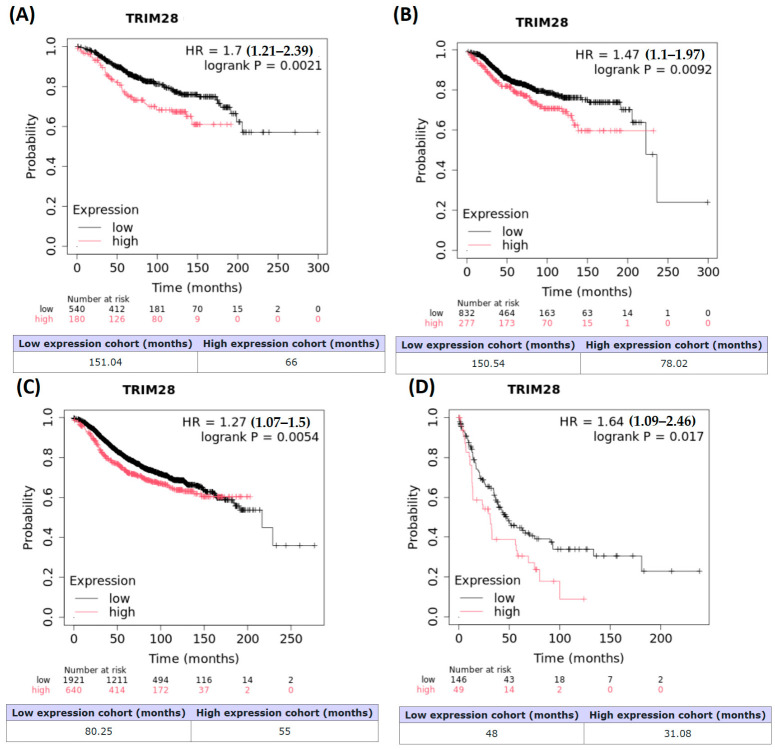
Kaplan–Meier survival curves evaluating the prognostic significance of TRIM28 mRNA expression in ERα-positive breast cancer patients. (**A**) Overall survival (OS); (**B**) distant metastases-free survival (DMFS); (**C**) relapse-free survival (RFS); (**D**) post-progression survival (PPS). Patients were stratified by upper quartile TRIM28 expression. Log-rank *p*-values < 0.05 were considered statistically significant.

The prognostic value of TRIM28 in ERα-positive breast cancer was explored by utilising the KM plotter website, in which four different parameters were used to assess prognosis. The results showed that higher TRIM28 significantly correlates with lower OS (Figure 2A), worse DMFS (Figure 2B), worse RFS (Figure 2C), and worse PPS (Figure 2D) in ERα-positive breast cancer.

### 3.3. TRIM28 mRNA Correlates Positively with Tumour Size, Neoplasm Histologic Grade, Nottingham Prognostic Index, and TMB in ERα-Positive Breast Cancer Patients and Negatively with Hormone Therapy in ERα-Positive Breast Cancer

**Figure 3:**
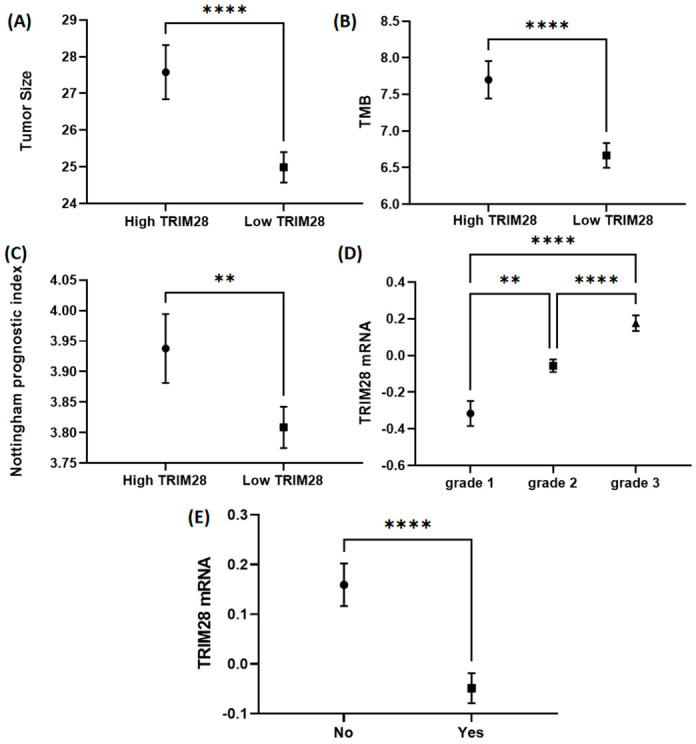
Association between TRIM28 mRNA expression and clinical parameters in ERα-positive breast cancer patients from the METABRIC dataset. (**A**) Tumour size; (**B**) tumour mutational burden (TMB); (**C**) Nottingham Prognostic Index (NPI); (**D**) neoplasm histologic grade; (**E**) hormone therapy status (received vs. not received). ** *p* < 0.01; **** *p* < 0.0001.

The METABRIC dataset in the cBio Cancer Genomics Portal was explored to further validate our observation that TRIM28 mRNA correlates with worse prognosis in ERα-positive breast cancer. Significantly higher tumour size and TMB were observed in high TRIM28 mRNA compared to low TRIM28 mRNA (Figure 3A,B). Moreover, a higher Nottingham Prognostic Index score was observed in high TRIM28 mRNA compared to low TRIM28 mRNA (Figure 3C). Significant positive correlation between TRIM28 and neoplasm histologic grades was observed, in which higher TRIM28 mRNA was in grade 3 compared to grades 2 and 1 (Figure 3D). Additionally, significantly lower TRIM28 mRNA was observed in ERα-positive breast cancer patients who received hormone therapy compared to patients who did not receive hormone therapy (Figure 3E).

### 3.4. TRIM28 Expression Is an Independent Predictor of OS in ERα-Positive Breast Cancer

Multivariate Cox proportional hazards regression analysis was carried out using the METABRIC dataset (n = 1356 ERα-positive breast cancer patients with complete data; 791 events) to evaluate whether TRIM28 expression constitutes an independent prognostic factor beyond established variables. The primary multivariate model adjusted for tumour size, TMB, hormone therapy status, and age at diagnosis, with stratification on neoplasm histological grade to address proportional hazards (PH) violation. Nottingham Prognostic Index (NPI) was excluded from the primary multivariate model because it is a composite metric mathematically derived from tumour size, lymph node stage, and histological grade (NPI = 0.2 × tumour size + lymph node stage + grade), and its inclusion alongside its constituent variables introduces multicollinearity that distorts coefficient estimates for grade. High TRIM28 expression remained a statistically significant and independent predictor of OS in the grade-stratified primary model (HR = 1.205, 95% CI: 1.031–1.409, *p* = 0.0194) as shown in Table 2. Tumour size (HR = 1.014, 95% CI: 1.010–1.018, *p* < 0.0001) and age at diagnosis (HR = 1.045 per year, 95% CI: 1.038–1.053, *p* < 0.0001) were independently associated with OS, whereas hormone therapy status (HR = 1.153, 95% CI: 0.975–1.363, *p* = 0.0958) and TMB (HR = 1.000, 95% CI: 0.986–1.015, *p* = 0.9814) no longer reached statistical significance after adjustment for age, indicating that part of their previously observed association with OS was confounded by age. The model showed improved discriminatory performance after adding age (C-index = 0.657, compared with 0.607 in the original model). Schoenfeld residuals confirmed that TRIM28, tumour size, hormone therapy, and TMB satisfied the PH assumption in the age-adjusted, grade-stratified model; however, age at diagnosis itself violated the PH assumption when modelled as a continuous covariate (*p* = 0.0005). As a sensitivity analysis, age was instead stratified as tertiles alongside grade, which resolved the PH violation and yielded a consistent result for TRIM28 (HR = 1.190, 95% CI: 1.016–1.394, *p* = 0.0312; C-index = 0.595; Supplementary Table S2). To address concerns regarding the choice of expression cutoff, sensitivity analyses were performed using alternative TRIM28 dichotomisation thresholds (median, tertile, upper quartile, top decile); the prognostic effect was directionally consistent across cutoffs, with the upper quartile threshold yielding the strongest signal (Supplementary Table S2). When TRIM28 was modelled as a continuous z-score variable, a positive but non-significant trend was observed (HR = 1.075 per unit, 95% CI: 0.998–1.158, *p* = 0.058), indicating that the prognostic signal is concentrated in the upper tail of TRIM28 expression. PAM50 subtype-specific subgroup analyses showed an independent association of high TRIM28 with worse OS in Luminal A (n = 638, events = 337; HR = 1.37, 95% CI: 1.08–1.74, *p* = 0.009), and a directionally consistent but non-significant association in Luminal B (n = 435, events = 284; HR = 1.21, 95% CI: 0.93–1.58, *p* = 0.155). The originally specified Cox model that included NPI alongside its component variables (tumour size and grade) is reported as a sensitivity analysis in Supplementary Table S3 for completeness; in that specification, TRIM28 retained independent prognostic significance (HR = 1.333, 95% CI: 1.139–1.560, *p* = 0.0003), but the histological grade coefficient was distorted (HR = 0.806, *p* = 0.0085), reflecting the collinearity between grade and NPI noted above. Across all model specifications and cutoffs, the prognostic association of TRIM28 with worse OS was robust.

**Table 1:** The clinical and demographic characteristics of the patient cohorts used in this study.

Characteristic	Bc-GenExMiner	METABRIC (ERα-Positive)	TCGA-BRCA (ERα-Positive)
Total patients (n)	530 (ERα+)	1535	372
ERα-negative (n)	187	0 (excluded)	0 (excluded)
Tumour-adjacent tissue (n)	89	—	—
Normal breast tissue (n)	92	—	—
Histologic Grade 1 (n)	—	162 (10.6%)	Not available
Histologic Grade 2 (n)	—	721 (47.0%)	Not available
Histologic Grade 3 (n)	—	582 (37.9%)	Not available
Grade missing (n)	—	70 (4.6%)	Not available
Tumour Stage/T-stage 1 (n)	—	391 (25.5%)	104 (28.0%)
Tumour Stage/T-stage 2 (n)	—	617 (40.2%)	209 (56.2%)
Tumour Stage/T-stage 3 (n)	—	68 (4.4%)	43 (11.6%)
Tumour Stage/T-stage 4 (n)	—	8 (0.5%)	16 (4.3%)
Stage missing/0 (n)	—	451 (29.4%)	0
Median tumour size (mm)	—	22	—
Median NPI	—	4.04	—
Median age (years)	—	—	60
Median TMB (mut/Mb)	—	6.54	1.00
Hormone therapy: Yes (n)	—	1019 (66.4%)	—
Hormone therapy: No (n)	—	412 (26.8%)	—
Hormone therapy: missing (n)	—	104 (6.8%)	—
Median follow-up (months)	—	—	33.1
Deaths/events (n)	—	846	56
Database	Bc-GenExMiner v4.1	cBioPortal	cBioPortal (Firehose Legacy)
Platform	Microarray	Microarray	RNA-seq

NPI, Nottingham Prognostic Index; TMB, tumour mutational burden. All Bc-GenExMiner analyses were restricted to the ERα-positive population. The gene correlation analysis in Table 3 was performed on the ERα-positive population using the Bc-GenExMiner gene-correlation module (n = 3685 informative samples), whereas the differential expression analysis in Figure 1 used the ERα-positive cohort available in the expression module (n = 530); the two modules draw on different numbers of contributing samples.

**Table 2:** Multivariate Cox proportional hazards regression analysis of overall survival in ERα-positive breast cancer patients from the METABRIC dataset (n = 1356; events = 791). TRIM28 mRNA expression was dichotomised at the 75th percentile z-score (cutoff = 0.614). The primary multivariate model adjusted for tumour size, TMB, hormone therapy status, and age at diagnosis, with stratification on histologic grade. Nottingham Prognostic Index was excluded due to its mathematical collinearity with grade and tumour size; the original specification including NPI is reported as a sensitivity analysis in Supplementary Table S3. Statistically significant associations (*p* < 0.05) are indicated in bold.

Variable	HR	95% CI	*p*-Value
TRIM28 (high vs. low)	1.21	1.031–1.409	0.0194
Tumour size	1.014	1.010–1.018	<0.0001
TMB	1.000	0.986–1.015	0.981
Hormone therapy	1.153	0.975–1.363	0.0958
Age at diagnosis (per year)	1.045	1.038–1.053	<0.0001

HR, hazard ratio; CI, confidence interval; TMB, tumour mutational burden. The primary model is grade-stratified; histologic grade and Nottingham Prognostic Index are not shown as model coefficients (grade is a stratification variable; NPI is excluded due to collinearity). C-index = 0.657. A sensitivity analysis stratifying age as tertiles (to address a Schoenfeld-residual PH violation for age as a continuous covariate) gave a consistent result for TRIM28 (HR = 1.190, 95% CI: 1.016–1.394, *p* = 0.0312; C-index = 0.595; Supplementary Table S2). Sensitivity analyses (alternative TRIM28 cutoffs, continuous TRIM28, PAM50 subgroup analyses, NPI-included specification) are presented in Supplementary Tables S2 and S3.

### 3.5. Independent Validation of TRIM28 Prognostic Significance in TCGA-BRCA

**Figure 4:**
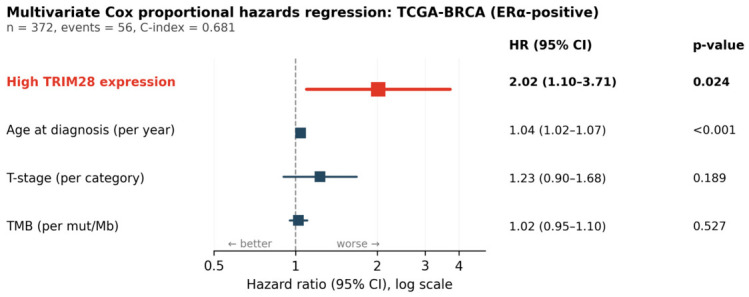
Independent validation of TRIM28 prognostic significance in the TCGA-BRCA Firehose Legacy ERα-positive cohort using multivariate Cox proportional hazards regression. A forest plot showing the multivariate-adjusted hazard ratios (HR) and 95% confidence intervals for high TRIM28 expression (upper quartile cutoff, z-score ≥ 0.807) and the covariates included in the model (age at diagnosis, T-stage, and tumour mutational burden [TMB]) as predictors of overall survival in TCGA-BRCA ERα-positive patients (n = 372 with complete data; events = 56). High TRIM28 expression was independently associated with worse overall survival (HR = 2.02, 95% CI: 1.10–3.71, *p* = 0.024). The model C-index was 0.681. The primary variable of interest (high TRIM28 expression) is shown in red; all other covariates are shown in black. The dashed vertical line indicates HR = 1.0 (no effect); the x-axis is shown on a logarithmic scale.

To independently validate the prognostic association of TRIM28 identified in the METABRIC cohort, multivariate Cox proportional hazards regression analysis was performed in the independent TCGA-BRCA cohort (Firehose Legacy). The analysis was restricted to patients with ERα-positive disease (by IHC), available TRIM28 mRNA expression data (RNA-seq z-scores relative to diploid samples), and complete OS follow-up data (n = 372 patients with 56 events; median follow-up = 33.1 months). TRIM28 expression was dichotomised at the upper quartile (z-score ≥ 0.807) to maintain methodological consistency with the METABRIC primary analysis. The multivariate model adjusted for age at diagnosis, T-stage, and TMB; histologic grade was not available for TCGA-BRCA patients and could therefore not be included as a covariate. After multivariate adjustment, high TRIM28 expression was independently associated with significantly worse OS in TCGA-BRCA ERα-positive patients (HR = 2.02, 95% CI: 1.10–3.71, *p* = 0.024; C-index = 0.681), confirming the prognostic association of TRIM28 identified in METABRIC (Figure 4). Notably, the magnitude of the effect was greater in TCGA-BRCA than in METABRIC, which may reflect differences between the cohorts in patient age distribution, follow-up duration, and the nature of recorded events. Of note, in the absence of multivariate adjustment, the prognostic association of TRIM28 in TCGA-BRCA did not reach statistical significance (univariate HR = 1.49, 95% CI: 0.83–2.68, *p* = 0.181), highlighting the importance of accounting for established prognostic covariates such as age and tumour size when evaluating the independent contribution of candidate biomarkers. As in METABRIC, sensitivity analyses across alternative TRIM28 cutoffs in TCGA-BRCA confirmed that the prognostic signal was concentrated in the upper expression tail (Supplementary Table S4).

### 3.6. TRIM28 mRNA Correlates Negatively with Numerous Gene Markers of Immune Cells in ERα-Positive Breast Cancer

**Figure 5:**
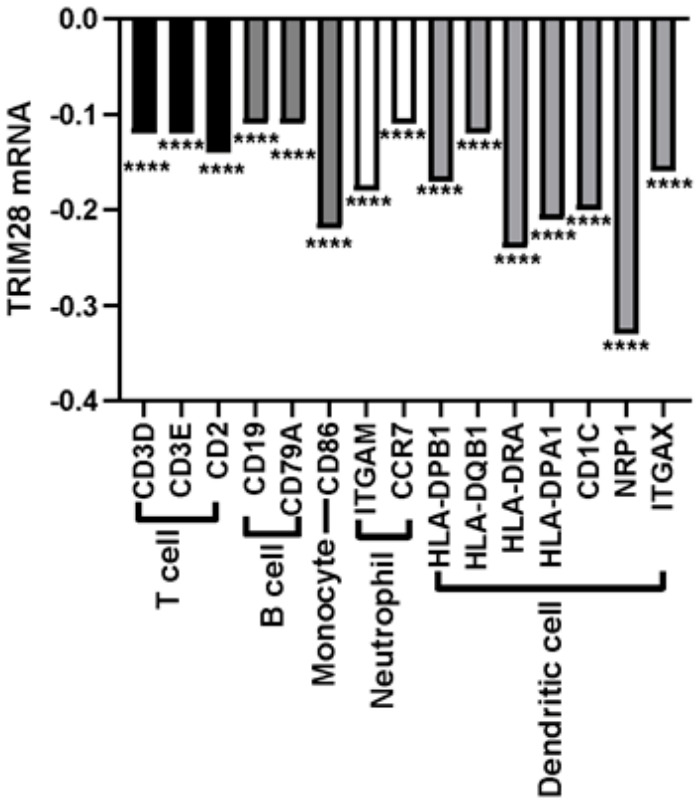
Pearson’s correlation coefficients between TRIM28 mRNA expression and gene markers of immune cell populations in ERα-positive breast cancer patients (n = 530), assessed using Bc-GenExMiner v4.1. Immune cell markers include T cells, B cells, monocytes, dendritic cells, and neutrophils. **** *p* < 0.0001.

Given the fact that the infiltration of immune cells in breast cancer correlates with better prognosis [27,28,29] and that TRIM28 correlates with worse prognosis in ERα-positive breast cancer, this prompted the investigation of the type of immune cells infiltrated in ERα-positive breast cancer. Significant negative correlations were observed between TRIM28 and numerous gene markers of T cells, B cells, monocytes, neutrophils, and dendritic cells in ERα-positive breast cancer patients (Figure 5).

### 3.7. Identifying the Top 20 Genes That Correlate Positively with TRIM28 in ERα-Positive Breast Cancer

**Figure 6:**
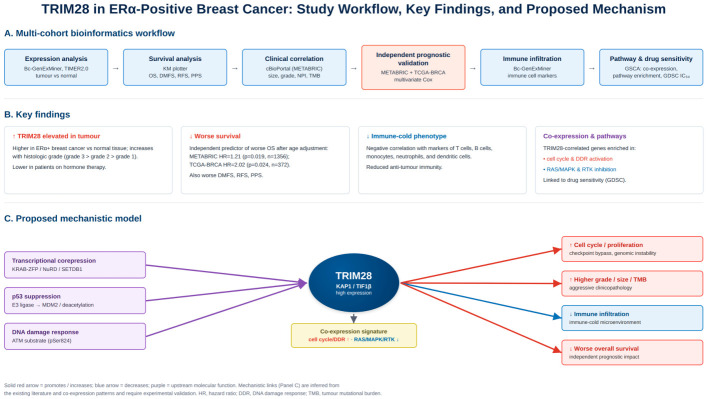
An integrated overview of the study design, key findings, and proposed mechanistic model for TRIM28 in ERα-positive breast cancer. (**A**) The multi-cohort bioinformatics workflow, comprising expression analysis (Bc-GenExMiner, TIMER2.0), survival analysis (Kaplan–Meier plotter; OS, DMFS, RFS, PPS), clinical correlation (cBioPortal/METABRIC), independent prognostic validation by multivariate Cox regression (METABRIC and TCGA-BRCA), immune infiltration analysis (Bc-GenExMiner), and pathway and drug-sensitivity analysis (GSCA, GDSC). (**B**) Summary of key findings: TRIM28 is elevated in ERα-positive tumours and increases with histologic grade; high TRIM28 expression independently predicts worse overall survival (METABRIC HR = 1.21, *p* = 0.019, adjusted for age; TCGA-BRCA HR = 2.02, *p* = 0.024); TRIM28 is associated with an immune-cold phenotype; and TRIM28-correlated genes are enriched in cell cycle and DNA damage response (DDR) activation signatures and RAS/MAPK and RTK pathway inhibition signatures. (**C**) The proposed mechanistic model in which the established molecular functions of TRIM28 (transcriptional corepression via KRAB-ZFP/NuRD/SETDB1, suppression of p53 through its E3 ligase activity, and participation in the ATM-dependent DNA damage response) converge to promote cell cycle progression, an aggressive clinicopathological phenotype, an immune-cold microenvironment, and worse survival. The red arrows indicate promotion/increase; the blue arrows indicate decrease; purple denotes upstream molecular functions. The mechanistic links shown in panel C are inferred from the existing literature and from co-expression patterns and require experimental validation. HR, hazard ratio; DDR, DNA damage response; TMB, tumour mutational burden.

The top 20 genes that correlate significantly and positively with TRIM28 in ERα-positive breast cancer were investigated to assess the pathways in which TRIM28 exerts its effect on prognosis (an integrated overview of the study design, key findings, and proposed mechanistic model is provided in Figure 6). The top genes that showed significant positive Pearson’s correlation (*p*-value < 0.0001, correlation coefficient > 0.5) with TRIM28 in ERα-positive breast cancer were selected (Table 3).

**Table 3:** The top 20 genes positively correlated with TRIM28 mRNA in ERα-positive breast cancer patients. The analysis was performed on the ERα-positive population using the Bc-GenExMiner gene-correlation module (n = 3685 informative samples); genes with Pearson’s correlation coefficient > 0.5 and *p* < 0.0001 were selected.

Symbol	Pearson’s Correlation Coefficient	*p*-Value	No. Patients
*ZBTB45*	0.7493	<0.0001	3685
*UBE2M*	0.6966	<0.0001	3685
*HSPBP1*	0.6746	<0.0001	3685
*RUVBL2*	0.6648	<0.0001	3685
*PRPF31*	0.6337	<0.0001	3685
*U2AF2*	0.6326	<0.0001	3685
*EPN1*	0.6126	<0.0001	3685
*MED25*	0.6116	<0.0001	3685
*CNOT3*	0.6075	<0.0001	3685
*PPP2R1A*	0.6002	<0.0001	3685
*ZNF444*	0.5999	<0.0001	3685
*ZNF787*	0.5993	<0.0001	3685
*ZNF446*	0.5968	<0.0001	3685
*TSEN34*	0.5957	<0.0001	3685
*ZNF579*	0.5952	<0.0001	3685
*SCAF1*	0.5925	<0.0001	3685
*ZNF324*	0.5909	<0.0001	3685
*SLC27A5*	0.5847	<0.0001	3685
*PTOV1*	0.5832	<0.0001	3685
*ZNF324B*	0.5719	<0.0001	3685

Gene correlation analysis was performed across all breast cancer patients available in Bc-GenExMiner (n = 3685) to maximise statistical power for the identification of genes robustly co-expressed with TRIM28. This cohort is distinct from the ERα-positive subset (n = 530) used for subtype-specific expression analysis. All correlations with Pearson r > 0.5 and *p* < 0.0001 are shown.

### 3.8. Genes Co-Expressed with TRIM28 in ERα-Positive Breast Cancer Are Enriched in Cell Cycle and DNA Damage Response Activation Signatures and RAS/MAPK and RTK Pathway Inhibition Signatures

**Figure 7:**
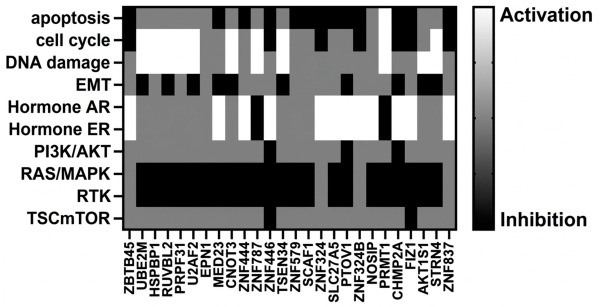
Heat map generated by GSCA showing cancer-related pathway activation and inhibition signatures enriched among genes positively correlated with TRIM28 in ERα-positive breast cancer patients. Note: Not all genes listed in Table 3 appear in this analysis, as GSCA pathway analysis is restricted to genes annotated within its curated cancer pathway gene sets.

To assess the enrichment of genes co-expressed with TRIM28 in cancer-related pathway signatures in ERα-positive breast cancer, genes that showed a significant positive correlation with TRIM28 expression were selected for pathway analysis. It was observed that genes significantly correlated with TRIM28 expression in ERα-positive breast cancer patients were enriched in RAS/MAPK and RTK pathway inhibition signatures and cell cycle and DNA damage response activation signatures (Figure 7). Given that GSCA pathway analysis is restricted to genes annotated within its curated cancer pathway gene sets, not all genes listed in Table 3 appear in Figure 7.

### 3.9. TRIM28 Positively Correlates with Docetaxel Resistance and Methotrexate Sensitivity in Cancer Cell Lines

**Figure 8:**
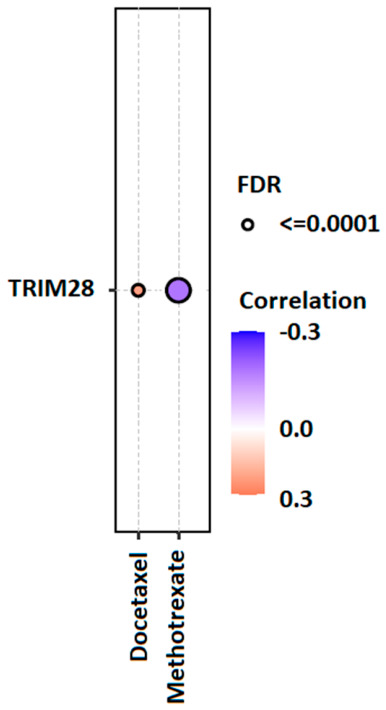
Correlation between TRIM28 mRNA expression and the IC_50_ of cancer drugs from the Genome of Drug Sensitivity in Cancer (GDSC) database, assessed using GSCA. Positive correlation indicates potential drug resistance; negative correlation indicates potential drug sensitivity. FDR < 0.0001.

Using the GDSC database, the correlation between TRIM28 and the IC_50_ of different breast cancer drugs was investigated. The expression of TRIM28 correlates positively with docetaxel IC_50_ in cancer cell lines, whereas it correlates negatively with methotrexate IC50 (Figure 8).

## 4. Discussion

The findings indicate that TRIM28 mRNA expression is significantly elevated in breast cancer tissues relative to tumour-adjacent and normal breast tissues. Furthermore, TRIM28 expression is markedly increased in ERα-positive breast cancer compared to ERα-negative breast cancer, suggesting a potential role for TRIM28 as a prognostic biomarker in ERα-positive breast cancer. These results are consistent with a prior study, which similarly reported elevated TRIM28 expression in breast cancer. However, the study did not show the potential role for TRIM28 as a prognostic biomarker in ERα-positive breast cancer [16]. Additionally, upregulation of TRIM28 compared to normal tissue has been documented in other malignancies, including prostate, gastric, ovarian, cervical, colorectal, glioma and lung cancer [12,30,31,32].

The prognostic significance of TRIM28 in ERα-positive breast cancer was further evaluated using the Kaplan–Meier plotter, which revealed a positive correlation between elevated TRIM28 expression and poorer prognosis in ERα-positive breast cancer, indicating its potential as a predictive biomarker for poor prognosis in this subtype. While the prognostic role of TRIM28 in ERα-positive breast cancer remains underexplored, the existing literature suggests an association between TRIM28 expression and reduced OS in breast cancer [16]. A similar pattern has been observed in other malignancies, in which TRIM28 expression has been shown to correlate positively with worse OS in lung adenocarcinoma, kidney renal papillary cell carcinoma, low-grade glioma cancer, liver hepatocellular carcinoma, and mesothelioma [33]. Our analysis revealed that TRIM28 correlates positively with tumour size in ERα-positive breast cancer, which has been documented in other malignancies, including hepatocellular carcinoma and triple-negative breast cancer [34,35].

Our multivariate Cox regression findings confirmed the independent prognostic value of TRIM28, as high TRIM28 expression independently predicted worse OS after adjustment for tumour size, TMB, hormone therapy status, and age at diagnosis (with stratification on histologic grade) in METABRIC (HR = 1.205, 95% CI: 1.031–1.409, *p* = 0.0194). Age at diagnosis was itself the strongest covariate in the model (HR = 1.045 per year, 95% CI: 1.038–1.053, *p* < 0.0001), and accounting for it attenuated, but did not eliminate, the prognostic association of TRIM28. This finding was independently validated in the TCGA-BRCA Firehose Legacy ERα-positive cohort using multivariate Cox regression adjusted for age, T-stage, and TMB (HR = 2.02, 95% CI: 1.10–3.71, *p* = 0.024). Of note, the prognostic association of TRIM28 in TCGA-BRCA was not statistically significant in univariate analysis (HR = 1.49, *p* = 0.181), illustrating that adjustment for established prognostic covariates—particularly age, which is strongly associated with overall survival—is essential for evaluating the independent contribution of TRIM28. The larger effect size observed in TCGA-BRCA compared to METABRIC may reflect the shorter follow-up duration of TCGA, which results in deaths that occur on average earlier and may therefore correspond to more biologically aggressive disease. PAM50 subtype-specific subgroup analyses within METABRIC further demonstrated that the prognostic association of TRIM28 was preserved in Luminal A breast cancer (HR = 1.37, *p* = 0.009) and was directionally consistent in Luminal B (HR = 1.21, *p* = 0.155). These findings are consistent with a published pan-cancer analysis identifying TRIM28 as an independent prognostic predictor across multiple cancers [33], and extend this observation specifically to the ERα-positive breast cancer subtype. Collectively, our multi-cohort multivariate findings nominate TRIM28 as an independent prognostic biomarker for worse OS in ERα-positive breast cancer patients.

Considering that TRIM28 positively correlates with DMFS, higher tumour grades, and Nottingham Prognostic Index in ERα-positive breast cancer, it is suggested that TRIM28 may contribute to invasion and metastasis in this subtype. These results are consistent with a prior study, which similarly reported that elevated TRIM28 expression correlates with metastasis in breast cancer [33]. It has been suggested that TRIM28 promotes the migration and invasion of breast cancer cells by activating the AKT/GSK3β signalling pathway [14]. Although several reports explored the role of TRIM28 in mediating metastasis in breast cancer, the studies did not investigate the role of TRIM28 in the ERα-positive subtype.

To further validate our observation that TRIM28 correlates with poor prognosis in ERα-positive breast cancer, the infiltration of immune cells was investigated, as infiltration correlates with better prognosis in breast cancer [27,28,29]. The significant negative correlations between TRIM28 and several immune cells agree with our previous observation that TRIM28 correlates with poor prognosis in ERα-positive breast cancer. Our observations agree with a published report showing that TRIM28 is negatively associated with the levels of most immune cells, including B cells, CD8^+^ T cells, macrophages, neutrophils and dendritic cells in lung adenocarcinoma patients [36]. Moreover, another study demonstrated that silencing TRIM28 enhanced the infiltration of CD8^+^ T cells in prostate cancer [37].

Our results indicated that genes co-expressed with TRIM28 are enriched in DNA damage response and cell cycle activation signatures and RAS/MAPK and RTK pathway inhibition signatures in ERα-positive breast cancer patients. These observations suggest that inhibiting the RAS/MAPK signalling pathway during DNA damage response might contribute to ERα-positive breast cancer progression. A published report supports our observation, demonstrating that inhibition of the MEK-ERK signalling pathway, the core segment of the broader MAPK signalling pathway, during the DNA damage response promotes tumour progression by bypassing DNA damage response checkpoints, leading to somatic mutation accumulation and cancer proliferation [38]. Moreover, several reports showed that inhibiting the RTK signalling pathway during DNA damage response promotes the invasion and progression of different types of cancers, including glioblastoma, head and neck cancer, and triple-negative breast cancer [39,40,41].

Beyond these pathway-level associations, the established molecular functions of TRIM28 provide a plausible mechanistic basis for its enrichment among cell cycle and DNA damage response (DDR) genes in ERα-positive breast cancer. TRIM28 is a multifunctional scaffold protein that integrates transcriptional regulation, chromatin remodelling, and the DNA damage response [42]. As a transcriptional corepressor, TRIM28 binds Krüppel-associated box zinc-finger proteins (KRAB-ZFPs) and recruits the nucleosome remodelling and deacetylase (NuRD) complex and the histone methyltransferase SETDB1, promoting heterochromatin formation and gene silencing [43]; this chromatin-regulatory activity is closely coupled to cell cycle progression and genome stability. Through its intrinsic E3 SUMO and ubiquitin ligase activities, TRIM28 also negatively regulates the tumour suppressor p53, both by facilitating MDM2-mediated p53 degradation and by promoting p53 deacetylation, thereby attenuating p53-dependent cell cycle arrest and apoptosis and permitting continued proliferation of tumour cells [9]. In the context of the DDR, TRIM28 is a direct substrate of the ataxia-telangiectasia mutated (ATM) kinase, which phosphorylates TRIM28 at serine 824 in response to DNA double-strand breaks; this phosphorylation relaxes chromatin to facilitate repair and modulates DDR checkpoint signalling [44]. Collectively, these functions position TRIM28 as a node that simultaneously suppresses p53-mediated checkpoint control, remodels chromatin to support proliferation, and participates in DDR signalling—mechanisms that are coherent with the cell cycle and DDR activation signatures enriched among TRIM28-correlated genes in the present study. We propose that, in ERα-positive breast cancer, elevated TRIM28 expression may drive a more proliferative and genomically unstable phenotype through these coupled transcriptional and DDR functions, which would be consistent with the observed associations between high TRIM28 expression and higher histologic grade, larger tumour size, elevated TMB, reduced immune infiltration, and worse survival. We emphasise, however, that these mechanistic links are inferred from the existing literature and from co-expression patterns, and that direct functional validation—for example, through TRIM28 knockdown or overexpression in ERα-positive cell lines coupled with assessment of cell cycle progression, p53 pathway activity, and DDR markers—will be required to establish causality.

Our analysis showed that TRIM28 correlates positively with docetaxel IC_50_, suggesting that higher TRIM28 mRNA expression may be associated with docetaxel resistance. Hence, clinicians should be aware of potential docetaxel resistance when using this drug in breast cancer patients with high TRIM28 expression and might consider alternative drugs. Moreover, our analysis showed that TRIM28 expression negatively correlates with methotrexate IC_50_, suggesting that patients with high TRIM28 expression may be more sensitive to methotrexate treatment. Thus, clinicians should exercise caution with dosing when administering methotrexate to patients with high TRIM28 expression. Furthermore, our findings indicate that ERα-positive breast cancer patients treated with hormone therapy exhibited significantly reduced TRIM28 expression relative to those not receiving hormone therapy. Consequently, these results suggest that clinicians may consider hormone therapy as a targeted treatment strategy for ERα-positive breast cancer patients presenting with elevated TRIM28 expression. Given the positive correlation between TRIM28 expression and TMB in ERα-positive breast cancer patients, and evidence that elevated TMB is associated with improved responsiveness to immunotherapy [45], clinicians may contemplate the use of immunotherapy as a treatment option for ERα-positive breast cancer patients exhibiting high TRIM28 expression.

Although the aforementioned findings highlight the merits of bioinformatics-based approaches, several limitations warrant consideration. In particular, the precise mechanisms through which TRIM28 influences prognosis in ERα-positive breast cancer require further in vitro assays including TRIM28 knockdown and overexpression in ERα-positive breast cancer cell lines. Moreover, in vivo animal models should be carried out to confirm the findings. Batch effect correction across databases was not applied and potential database-specific biases in Bc-GenExMiner and KMplot may influence our findings. Another limitation of the study is that drug-sensitivity findings are based on cell line IC50 data from the GDSC database and require clinical validation before informing treatment decisions. It is further noted that the multivariate Cox model for the TCGA-BRCA validation cohort could not include histologic grade as a covariate because grade information is not curated in TCGA-BRCA; the model was therefore adjusted for age, T-stage, and TMB only. The TCGA-BRCA cohort also has shorter follow-up than METABRIC (median 33 vs. 122 months), which limits statistical power for long-term survival comparisons. The C-index of the multivariate models (0.61 in METABRIC, 0.68 in TCGA-BRCA) indicates moderate discriminatory performance, consistent with the inherent heterogeneity of ERα-positive breast cancer; TRIM28 should therefore be considered as a candidate biomarker contributing prognostic information beyond established clinicopathological variables, rather than as a standalone predictor.

## 5. Conclusions

In conclusion, our findings demonstrate that TRIM28 expression is significantly elevated in breast cancer tissues compared to normal breast tissue, with notably higher expression observed in histologic grade 3 versus grades 1 and 2 in ERα-positive breast cancer patients. Multivariate Cox proportional hazards regression confirmed that TRIM28 is an independent prognostic factor in ERα-positive breast cancer (HR = 1.205, 95% CI: 1.031–1.409, *p* = 0.0194, after adjustment for age; METABRIC, n = 1356), and was independently validated in the TCGA-BRCA Firehose Legacy ERα-positive cohort using multivariate Cox regression (HR = 2.02, 95% CI: 1.10–3.71, *p* = 0.024; n = 372). Furthermore, TRIM28 expression exhibits a positive correlation with worse OS, DMFS, RFS, and PPS in ERα-positive breast cancer patients. These results indicate that TRIM28 may serve as a promising biomarker for worse prognosis in ERα-positive breast cancer. Genes co-expressed with TRIM28 in ERα-positive breast cancer are enriched in cell cycle and DNA damage response activation signatures, and in RAS/MAPK and RTK pathway inhibition signatures, which provides a molecular basis for future mechanistic investigation. The observation that ERα-positive patients receiving hormone therapy have lower TRIM28 expression further suggests that TRIM28 may represent a therapeutically modifiable target in this subtype. Despite the insights provided by these bioinformatics analyses, further mechanistic studies utilising in vitro assays and in vivo animal models are essential to rigorously elucidate TRIM28’s role in mediating invasion and metastasis in ERα-positive breast cancer.

## Data Availability

The data presented in this study are available from publicly accessible databases. The METABRIC data were obtained from the cBio Cancer Genomics Portal (http://cbioportal.org). Kaplan–Meier survival analyses were performed using the Kaplan–Meier Plotter (http://kmplot.com/analysis/). Gene expression and immune cell infiltration data were retrieved from Bc-GenExMiner version 4.1 (http://bcgenex.ico.unicancer.fr) and TIMER2.0 (http://timer.cistrome.org/). Pathway and drug-sensitivity analyses were conducted using GSCA (http://bioinfo.life.hust.edu.cn/GSCA/#/). All databases were accessed on 12 October 2025. No new primary data were generated in this study. The R code used for multivariate Cox proportional hazards regression analysis is available from the corresponding author upon reasonable request.

## Supplementary Materials

The following supporting information can be downloaded at https://www.mdpi.com/article/10.3390/biomedicines14071523/s1. Table S1: METABRIC dataset containing clinical and molecular data for ERα-positive breast cancer patients used in this study. Table S2: Sensitivity analyses for the METABRIC primary multivariate Cox model, including alternative TRIM28 dichotomisation cutoffs (median, tertile, upper quartile, top decile), TRIM28 modelled as a continuous z-score variable, and PAM50 subtype-specific subgroup analyses (Luminal A and Luminal B). Table S3: Original (NPI-included) multivariate Cox proportional hazards regression analysis of overall survival in ERα-positive breast cancer patients from the METABRIC dataset, retained as a sensitivity analysis. Table S4: Sensitivity analyses for the TCGA-BRCA Firehose Legacy validation, including alternative TRIM28 dichotomisation cutoffs and univariate Cox regression results for comparison. Figure S1: A patient flow diagram illustrating the inclusion and exclusion criteria applied to the METABRIC dataset.

## Abbreviations

The following abbreviations are used in this manuscript:

TRIM28	Tripartite motif-containing 28
GSCA	Gene Set Cancer Analysis
Bc-GenExMiner	Breast Cancer Gene-Expression Miner
RTK	Receptor tyrosine kinase
KRAB	Krüppel-associated box
KAP1	KRAB-associated protein 1
TIF1β	Transcriptional intermediary factor 1β
EMT	Epithelial-to-mesenchymal transition
PPS	Post-progression survival
OS	Overall survival
DMFS	Distant metastases-free survival
RFS	Relapse-free survival
TMB	Tumour mutational burden
HR	Hazard ratio
CI	Confidence interval
NPI	Nottingham Prognostic Index
PH	Proportional hazards
GDSC	Genome of Drug Sensitivity in Cancer
METABRIC	Molecular Taxonomy of Breast Cancer International Consortium
TCGA	The Cancer Genome Atlas
TCGA-BRCA	The Cancer Genome Atlas Breast Cancer dataset
TIMER2.0	Tumor IMmune Estimation Resource version 2.0
LumA	Luminal A
LumB	Luminal B
FDR	False discovery rate
