# Supplementary material for: High TRIM28 Expression Defines an Aggressive, Immune-Cold Phenotype with Worse Survival Outcomes in ERα-Positive Breast Cancer
Source: Biomedicines. 2026 Jul 7;14(7):1523. doi: 10.3390/biomedicines14071523 (PMC13406493; doi:10.3390/biomedicines14071523)
Supplement: Supplementary file 1 [file biomedicines-14-01523-s001.zip › biomedicines-4382680-Supplementary Tables S2-S4.pdf]

Supplementary Materials

High TRIM28 Expression Defines an Aggressive, Immune-Cold Phenotype with Worse Survival Outcomes in ERα-Positive Breast Cancer

Supplementary Table S2

Sensitivity analyses for the METABRIC primary multivariate Cox model. The primary model adjusted for tumour size, TMB, and hormone therapy status, with stratification on histologic grade and exclusion of NPI to avoid multicollinearity. n = 1,356 ERα-positive breast cancer patients (events = 791).

Panel A. Alternative TRIM28 dichotomisation cutoffs (multivariate, grade-stratified)

Cutoff	Z-score	n high / n total	HR (95% CI)	p-value
Median (50%)	−0.010	678 / 1356	1.144 (0.991–1.320)	0.067
Tertile (66.7%)	0.350	452 / 1356	1.190 (1.026–1.379)	**0.021**
**Upper quartile (75%) — primary**	**0.609**	**339 / 1356**	**1.268 (1.084–1.482)**	**0.0029**
Top decile (90%)	1.154	136 / 1356	1.122 (0.901–1.399)	0.304

Panel B. TRIM28 modelled as continuous z-score (multivariate, grade-stratified)

Variable	HR per unit z-score (95% CI)	p-value
TRIM28 (continuous)	1.076 (0.999–1.159)	0.054

Panel C. PAM50 subtype-specific subgroup analyses (multivariate)

Subtype	n	Events	HR (95% CI)	p-value
**Luminal A**	638	337	**1.372 (1.083–1.737)**	**0.009**
Luminal B	435	284	1.213 (0.930–1.584)	0.155

HR, hazard ratio; CI, confidence interval. The prognostic effect of TRIM28 was directionally consistent across cutoffs, with the upper quartile threshold yielding the strongest signal. The continuous-variable analysis showed a borderline non-significant trend, indicating that the prognostic signal is concentrated in the upper expression tail. PAM50 subtype-specific analyses confirmed an independent association in Luminal A, with a directionally consistent but non-significant trend in Luminal B.

Supplementary Table S3

Original (NPI-included) multivariate Cox proportional hazards regression analysis of overall survival in ERα-positive breast cancer patients from the METABRIC dataset (n = 1,356; events = 791). This model is retained as a sensitivity analysis to demonstrate the robustness of the TRIM28 prognostic effect across model specifications. The histologic grade coefficient (HR = 0.806) is biologically counterintuitive due to multicollinearity between grade and NPI (NPI = 0.2 × tumour size + lymph node stage + grade); for this reason, NPI was excluded from the primary model presented in Table 2.

Variable	HR	95% CI	p-value

**TRIM28 (high vs low)**	**1.333**	**1.139–1.560**	**0.0003**
**Tumour size**	**1.013**	**1.009–1.017**	**<0.0001**
Histologic grade	0.806	0.687–0.947	0.009*
**Nottingham Prognostic Index**	**1.424**	**1.279–1.587**	**<0.0001**
TMB	1.001	0.987–1.016	0.850
Hormone therapy	1.085	0.906–1.300	0.376

HR, hazard ratio; CI, confidence interval; TMB, tumour mutational burden; NPI, Nottingham Prognostic Index. C-index = 0.635. *The negative coefficient for histologic grade is attributable to multicollinearity with NPI (which is mathematically derived from grade and tumour size); see Table 2 for the primary model in which NPI is excluded and grade is properly behaved.

Supplementary Table S4

Sensitivity analyses for the TCGA-BRCA Firehose Legacy validation cohort (n = 372 ERα-positive patients, 56 events). The primary multivariate Cox model adjusted for age at diagnosis, T-stage, and TMB. Histologic grade was not available in TCGA-BRCA and could not be included as a covariate.

Panel A. Alternative TRIM28 dichotomisation cutoffs (multivariate Cox)

Cutoff	Z-score	n high	HR (95% CI)	p-value
Median (50%)	0.019	186	1.262 (0.740–2.151)	0.392
Tertile (66.7%)	0.562	124	1.560 (0.865–2.814)	0.140
**Upper quartile (75%) — primary**	**0.807**	**93**	**2.019 (1.097–3.714)**	**0.024**
Top decile (90%)	1.605	38	1.413 (0.539–3.705)	0.482

Panel B. Univariate Cox regression (for comparison with multivariate primary model)

Model	HR (95% CI)	p-value
TRIM28 (univariate, upper quartile)	1.491 (0.831–2.676)	0.181
**TRIM28 (multivariate: + age, T-stage, TMB)**	**2.019 (1.097–3.714)**	**0.024**

HR, hazard ratio; CI, confidence interval. As in METABRIC, the prognostic signal is concentrated in the upper TRIM28 expression tail. The univariate analysis is non-significant, while the multivariate-adjusted model shows a strong, significant association — illustrating the importance of adjusting for established prognostic covariates (particularly age) when evaluating the independent contribution of TRIM28.
