# Supplementary figures and images for: High TRIM28 Expression Defines an Aggressive, Immune-Cold Phenotype with Worse Survival Outcomes in ERα-Positive Breast Cancer
Source: Biomedicines. 2026 Jul 7;14(7):1523. doi: 10.3390/biomedicines14071523 (PMC13406493; doi:10.3390/biomedicines14071523)

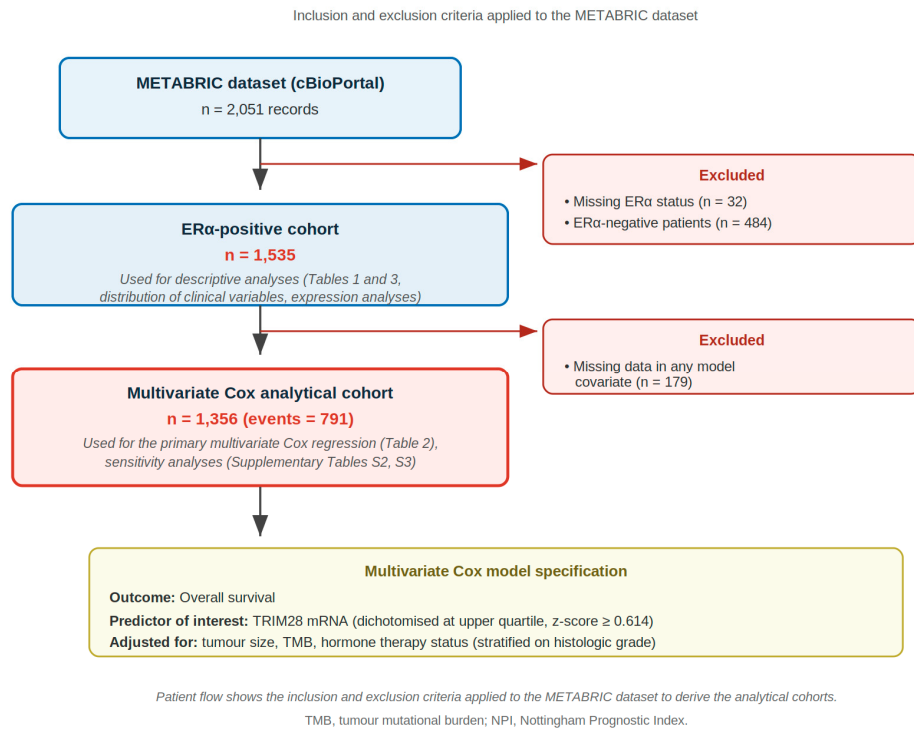

**Supplementary Figure S1.** METABRIC patient flow diagram.

Supplement: Supplementary file 1 [file biomedicines-14-01523-s001.zip › biomedicines-4382680-Supplementary Figure S1.pdf]
